# Impact of Active and Passive Hypoxia as Re-Warm-Up Activities on Rugby Players’ Performance

**DOI:** 10.3390/ijerph17082971

**Published:** 2020-04-24

**Authors:** Domingo Jesús Ramos-Campo, João Malta, Guillermo Olcina, Rafael Timón, Armando Raimundo, Pablo Tomas-Carus

**Affiliations:** 1 Faculty of Sports, UCAM, Catholic University San Antonio, 30107 Murcia, Spain; 2 Departamento de Desporto e Saúde, Escola de Ciências e Tecnologia, Universidade de Évora, 7000-645 Évora, Portugal; jbvm@uevora.pt (J.M.); ammr@uevora.pt (A.R.); ptc@uevora.pt (P.T.-C.); 3 Comprehensive Health Research Centre (CHRC), University of Évora, 7000-645 Évora, Portugal; 4 Faculty of Sport Sciences, University of Extremadura, 10003 Cáceres, Spain; golcina@unex.es (G.O.); rtimon@unex.es (R.T.)

**Keywords:** altitude, hypoxic training, jump, repeat sprint ability, sprint

## Abstract

The aim of this study was to analyse the effect of four types of re-warm-up (R-WU) activity, namely rest in normoxia (RN) at FiO_2_ = 20.9%, rest in hypoxia (RH) at FiO_2_ = 15%, activity (4 × 5 jumps/15 s) in normoxia (AN) and activity in hypoxia (AH) on physical performance. Ten elite male rugby players completed a 15-min warm-up followed by one of the 15-min randomized R-WU strategies. After R-WU, countermovement jump (CMJ), 20 m sprint and repeat sprint ability (RSA) tests were assessed. Compared to passive strategies (RN and RH), tympanic temperature was higher after active R-WU (AN and AH) (*p* = 0.016). Higher values of CMJ height (*p* = 0.037) and 20 m sprint (*p* = 0.02) were found in AH than in RN. In addition, mean RSA was lower (*p* = 0.008) in AH than in RN and RH. Blood lactate concentration was higher (*p* = 0.007) after RN and AN strategies than after AH. Muscle O_2_ saturation (*p* = 0.021) and total Hb (*p* = 0.042) were higher after AH than after the other three conditions and after RN, respectively. Therefore, an active R-WU under hypoxia could be useful to elite rugby players, once it had attenuated the decline in tympanic temperature during a 15-min period after warm-up, improving jump, sprint and RSA performance.

## 1. Introduction

Athletes are used to including a warm-up (WU) routine prior to training or competitive events with a view to optimize their subsequent performance [1]. The main effects of these WU exercises are highly dependent on an increase in body temperature [2], and it is well documented that WU routines promote an increase in blood flow through vasodilatation [3] and optimize metabolic reactions. In fact, these routines improve phosphate degradation and muscle glycolysis during exercise [4]. Moreover, WU routines are known to promote a decrease in joint and muscle resistance [2] and increase the nerve conduction rate [5]. In addition, WU sequences produce faster oxygen dissociation from haemoglobin [2], elevate baseline oxygen uptake (VO_2_) and increase the amplitude of primary VO_2_ response in subsequent exercise [6].

However, after ceasing exercise, body temperature declines rapidly, which reduces some of the benefits of the initial warm-up and impairs performance [7]. In fact, it has been demonstrated that every 1 °C decrease in muscle temperature results in a 3% decrease in muscle power [8]. Therefore, research suggests that WU routines should be conducted as close as possible to competitive events in order to have positive effects on performance. However, official requirements during some sports competitions (e.g., rugby matches) do not allow the requirements and optimal procedures of WU routines to be met. For example, at the professional level in rugby, there are some periods of inactivity, such as the pre-match transition protocol and the half-time break, and even when a player is punished for being admonished with a yellow card. In the official pre-match protocol, rugby players are required to return to the dressing room 15 min prior to the start of the game. During these periods of inactivity, body temperature can rapidly decrease and impair performance. Therefore, athletes and coaches tend to apply some re-warm-up routines (R-WU) during the period between the end of the WU and the start of the competitive action, closer to the start of the second half and during the 10-min punishment period, in order to minimize decremental effects on performance.

These R-WU routines are focused on prolonging the maintenance of body temperature during the period between the end of the WU and the start of competitive action [9], in the induction of post-activation potentiation (PAP) during half-time [10] and the punishment period, through passive and active R-WU strategies after warming up. Previous research with rugby players has shown that, in comparison with passive rest, the use of a passive heat jacket during the time between WU and the start of the match is an effective method of attenuating the post-warm-up decline in body temperature and improves jump performance and repeat sprint ability in professional rugby players [7]. Previous studies have classified rugby as a high-intensity, intermittent and collision sport, which requires players to repeat-sprint maximally (9‒50 m) and generate high levels of power [11]. These physiological factors can impair performance during the breaks and the periods of inactivity. Thus, active R-WU activities during the rest period after WU, such as plyometric and repeated change-of-direction exercises, attenuate losses in jump performance and sprint capacity in team sports [12], are also physiologically key factors in rugby.

Another hot topic is the use of passive exposure or exercise training in O_2_-deprived environments to minimize performance impairment during the rest period between WU and the start of the match after WU. Hence, active exercise under hypoxia (15% FiO_2_) as an R-WU activity seems to be an efficient activity to optimize swimming performance [13]. There are some positive effects produced by passive and active hypoxia that could be linked to WU responses. Active or passive hypoxia produces a specific molecular response arising from the oxygen-sensing pathways [14], improving O_2_ transport [15], glucose metabolism and lactate production to provide ATP [16]. In addition, exercise in O_2_-deprived environments produces greater reliance on the anaerobic metabolism [17] and a compensatory vasodilatation with an induced nitric oxide-dependent increase in muscle blood flow [18]; it also increases the baseline VO_2_ [19] more so than the same exercise in normoxia. Moreover, hypoxic exercise induces greater microvascular oxygen delivery to fast-twitch fibres [20] and higher motor unit recruitment [21] than the same exercise in normoxia. These metabolic and neuromuscular responses to hypoxic exposure could be allied to the WU effect, and for this reason, passive and active hypoxia could be used as a tool during an inactive phase to maintain the effects of warming up and optimize athletic performance. However, to the best of our knowledge, no studies have analysed the effects of performing an R-WU routine under hypoxia in team sports, specifically in rugby.

Therefore, the purpose of this study was to analyse the effect of four types of re-warm-up activities, namely (i) rest in normoxia (RN), (ii) rest in hypoxia (RH), (iii) activity in normoxia (AN) and (iv) activity in hypoxia (AH) on physical performance in elite rugby players. It was hypothesised that an active re-warm-up under hypoxic conditions will have a positive acute impact in vertical jump, sprint capacity and in the repeat sprint ability test.

## 2. Materials and Methods

### 2.1. Participants

Ten elite male rugby players (age: 22.9 ± 5.3 years; height: 180.2 ± 6.8 cm; weight: 89.2 ± 17.0 kg; playing experience: 8.6 ± 4.1 years) from the same team competing in the rugby union first division in Portugal participated in the present study. At the time that the study was conducted, players were taking part in three 90-min training sessions a week and two workouts in the gym, and playing in an official match at the weekend. None of the players reported having had any musculoskeletal disorder or exposure to altitude during the three months prior to the study. All participants were instructed to maintain their regular dietary consumption during the study and to avoid ingesting caffeine or alcohol for at least 24 h before each visit. A written consent was obtained from each volunteer in accordance with the Declaration of Helsinki. Our study was approved by the University of Évora Ethics Committee (Ref: 19007).

### 2.2. Design

A counterbalanced, repeated-measures cross-over design was used to determine whether different types of re-warm-up strategies affected rugby players’ performance. The rugby players completed the following four testing sessions with different re-warm-up routines: (i) RN with the fraction of inspired oxygen (FiO_2_) equal to 20.9%; (ii) RH with FiO_2_ equal to 15%; (iii) AN with FiO_2_ equal to 20.9%; and (iv) AH with FiO_2_ equal to 15%. The testing sessions under hypoxic conditions were performed in a normobaric chamber (CAT 430) using two generators (CAT-12, Colorado Altitude Training, Louisville, CO, USA). Re-warm-up phases under normoxia were also completed inside the normobaric chamber but the generators were turned off. The rugby players never knew whether the normobaric chamber was in a hypoxic or normoxic condition. The normobaric chamber was placed in a dressing room at a temperature of 22.0 ± 0.5 °C. All warm-ups and tests were carried out on an artificial-turf pitch (altitude: 290 m above sea level; temperature: 23.0 ± 0.7 °C).

### 2.3. Testing Procedure

Testing was carried out twice a week, during the usual training hours between 7 and 9 pm. Duration of all experiments was two weeks on consecutive Mondays and Thursdays (first testing on Monday, second testing on Thursday, third testing on Monday and fourth testing on Thursday). The rugby players had a rest day the day before each testing session. In each session, the players completed a standard active warm-up structured as follows: 2 min jogging, 4 × 30 m side run; dynamic stretching for main locomotive lower-limb muscles (2 × 10 hip adduction, 2 × 10 hip abduction, 2 × 10 butt kicks, 2 × 10 knee raises and 2 × 10 straight leg march), dynamic strength exercises (2 × 10 forward lunges and 2 × 10 deep squats); incremental intermittent sprints and agility runs (2 × 10 m pace, 2 × 20 m pace, 2 × 30 m with change of direction (180°) (COD) and pace, 1 × 20 m full pace and 1 × 30 m full pace with COD and two countermovement jumps (CMJ). The full-pace exercise (20 and 30 m with COD) and the best CMJ trial were used for data analysis. This warm-up protocol is based on previous studies [12] and the total time taken was 15 min.

**Figure 1:**
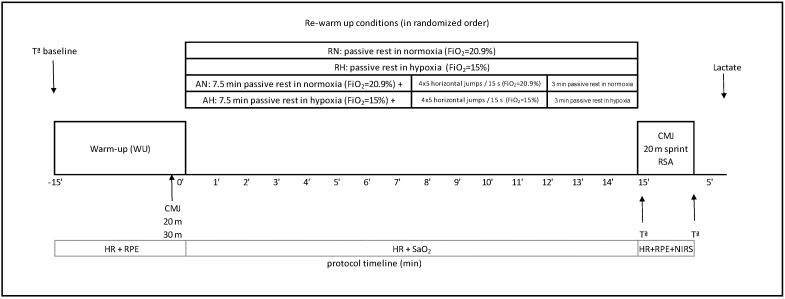
Schematic representation of experimental design. RN: rest in normoxia, AN: activity in normoxia; RH: rest in hypoxia; AH: activity in hypoxia, Tª: temperature; HR: heart rate; RPE: rating of perceived exertion; NIRS: near-infrared spectroscopy; SaO_2_: arterial oxygen saturation; WU: warm-up; RSA: repeat sprint ability test; CMJ: countermovement jump.

After the warm-up, participants rested passively for 15 min at 20.9 of FiO_2_ in RN or 15% of FiO_2_ in RH conditions. In both routines, the rugby players remained seated in the normobaric chamber (RN or RH) with minimal activity. In the other two conditions (AN and AH) they performed four sets of five maximum horizontal jumps (with free upper limbs and free degree of flexion of knees) and 15 s of passive recovery between sets exactly 7.5 min after the WU finished in the environmental conditions described above. After the jumps, the players rested for 3 min to complete a total of 15 min. The re-warm-up routines were distributed randomly among the participants. Finally, when the re-warm-up finished (15 min after the warm-up finished) the participants performed a CMJ test, a 20 m sprint test and a repeat sprint ability test (RSA) (Figure 1).

The tympanic temperature (Ttymp) was used as a proxy measure of deep body temperature. It was measured prior to the baseline WU, after the R-WU activity and immediately after the RSA test using a Braun ThermoScan IRT 4520 (Braun, Kronberg, Germany). The changes between the baseline temperature and after the R-WU and between baseline and after the RSA test were used for data analysis. The heart rate (HR) data were recorded using a Polar RS800 (Polar Electro, Kempele, Finland) monitor throughout the testing session and the mean and peak HR for each period were analysed. Ratings of perceived exertion (RPE) were determined using the 10-point Borg scale following the WU and the RSA test. Additionally, after the end of the R-WU, the SaO_2_ levels were measured using a pulse oximeter (Onyx, Nonin, Plymouth, MN, USA).

Countermovement jump heights were calculated using a contact platform (Ergotester, Globus, Codogne, Italy). Participants were asked to jump as high as possible with a rapid self-selected countermovement. The amplitude of knee flexion during the countermovement was also self-selected, and participants were asked to try and land close to the take-off point. Each participant performed two attempts, with 90 s of rest in between attempts. The best trial from each participant was used for data analysis.

After the CMJ test, a 20 m sprint test was carried out. The athlete started 0.5 m behind the start line, which was marked by a photocell (Witty, Microgate, Italy). Before starting, the athletes were instructed to run as fast as possible to the end of the 20 m course. After the 20 m sprint test, the RSA test was performed. The RSA test consisted of 10 × 30 m sprints with a 180° turn at the 15 m mark separated by 30 s of passive recovery [22]. The athlete started 0.5 m behind the start line, which was marked by a photocell. The best and mean sprint times were recorded as the performance indices. The fatigue index was calculated in accordance with previous studies [22].

In addition, a near-infrared spectroscope (NIRS) (Moxy, Fortiori Design, Minneapolis, Minnesota, USA) was used to determine the muscle oxygenation during the RSA test. The NIRS was positioned during the rest time of the re-warm-up activity on the participant’s dominant leg, on the vastus lateralis, halfway between the greater trochanter and lateral epicondyle of the femur. Prior to placement, this area was trimmed with an electric razor and cleaned with alcohol swabs. The muscle oxygen saturation (SmO_2_) and the total haemoglobin (THb) were recorded during the RSA test.

Finally, capillary blood samples (5 μL) for blood lactate concentration ({La-}) analysis were collected from a finger prick 5 min after the end of the RSA test and analysed using a Lactate Pro analyser (Lactate Pro, Arkay, Kyoto, Japan).

### 2.4. Statistical Analysis

Data analysis was performed using the statistical package SPSS v.24 (IBM, New York, NY, USA). Descriptive statistics with measures of central tendency and dispersion were used. The assumption of normality and homoscedasticity was verified with the Shapiro–Wilk Test. A one-way analysis of variance with repeated measures and Bonferroni post hoc was used to investigate differences between study variables. The effect size was calculated using Eta squared. For all procedures, a level of significance of *p* ≤ 0.05 was chosen.

## 3. Results

With respect to the warm-up variables, there were no differences among the four occasions that the rugby players were evaluated on RPE, mean and peak HR, 20 m and 30 m sprint, and CMJ height (Table 1).

With regard to the variables analysed during the four R-WU activities (Table 2), there was a significant effect on SaO_2_, with lower values for RH and AH than for RN and AN. In addition, there was a significant effect on the change (Δ) in Ttymp between baseline and after R-WU, showing significantly higher values in active (AN and AH) than in passive (RN and RH) R-WU strategies. However, no main effect was observed on mean and peak HR

In regards to performance and physiological variables after R-WU (Table 3), a significant effect was observed in the CMJ height and in the 20 m sprint, showing significantly higher values in AH than in RN. In addition, the mean RSA was significantly lower in AH than in passive R-WU activities (RN and RH). Furthermore, compared to AH, {La-} was significantly higher in normoxic conditions (RN and AN). Likewise, compared to AH, the mean SmO_2_ was significantly higher during the RSA test in the other three conditions. In addition, the mean muscle THb during RSA test was significantly higher in AH than in RN. However, no main effect was found on the mean and peak HR, RPE, the best sprint in the RSA test, the fatigue index and the changes in Ttymp after the RSA test.

## 4. Discussion

This study aimed to analyse the effects of including active or passive hypoxia in the period between the end of the WU and the start of the match, after a traditional warm-up, on CMJ performance, sprint capacity and in the RSA test in professional rugby players. The main findings indicate, for the first time, that including an active R-WU strategy under hypoxic conditions improves mean RSA performance in comparison with passive strategies (RN and RH). In addition, participants registered a significantly faster 20 m sprint, higher jump performance and lower mean muscle O_2_ saturation in the AH condition than in the RN condition.

The ability to maintain sprint speed decreases by 2.4% after 15 min of no activity [23]. However, players need to maintain the RSA performance over the course of a rugby game because it is a key factor in achieving successful performances [11]. Previous research with rugby players has shown the effectiveness of the use of passive and active R-WU strategies for attenuating impaired performance after warm-up [7]. However, our results are not in line with this previous study, because AN did not improve the RSA performance. On the other hand, other studies on rugby [24] that found similar results to those obtained in the present study showed no positive effect on RSA performance after an active R-WU strategy. According to previous studies [25], one possible reason for these controversial findings could be the low load of our chosen task where players only performed jumps with their body weight. Thus, this load may not be enough to create a potentiation effect, but the added stress generated by a low O_2_ environment (AH) could increase the stress of this exercise, leading to positive effects on the subsequent exercise as we reported in our results.

Prior anaerobic exercise improves muscle perfusion during subsequent exercise due to residual metabolic acidaemia produced in the initial exercise [26]. Two factors are likely involved in improving performance in the subsequent exercise: first, vasodilatation and elevated muscle blood flow at the start of the test; and second, the acidaemia-induced Bohr shift of the haemoglobin dissociation curve. This fact improves the diffusional gradient for O_2_ between the capillary and the muscle [26]. These physiological responses could be greater after exercise in hypoxia because this type of exercise produces a greater reliance on the anaerobic metabolism [17] and lactate production to provide synthetized ATP [16]. In addition, the limited O_2_ availability produced under hypoxia induces vasodilatation to increase the blood flow and the O_2_ delivery [22]. Therefore, one possible reason for the results obtained in the present study could be related to a higher stimulation of the anaerobic metabolism pathway during the AH strategy, which could improve the muscle perfusion during the subsequent RSA performance. Further support for a perfusion-related mechanism is provided by the muscle oxygenation data. In fact, during the RSA test, the mean values of Hb were significantly greater after AH than RH, which probably indicates a greater O_2_ delivery to the muscle during the test [27]. This finding is in accordance with a recent study on American football players, where the authors found an enhancement in muscle reoxygenation after a warm-up with local hypoxia (blood flow restriction) during a subsequent RSA test [27]. In addition, in the present study, there was a significantly higher muscle deoxygenation of the vastus lateralis during the RSA test after an AH re-warm-up strategy than in AN, RH and RN. This suggests that the muscle is capable of extracting more O_2_ during an RSA test in normoxia after AH re-warm-up.

In addition, it has been previously reported that active warm-up may allow subsequent tasks to begin with an elevated VO_2_, leaving more of the anaerobic capacity for later in the task. The initial sparing of the anaerobic capacity improves performance in tasks that require a significant anaerobic contribution [2]. This is supported by previous studies that reported a greater aerobic contribution [28], higher oxygen uptake, lower lactate concentration and higher blood pH [29] when tasks are preceded by an active exercise compared with no exercise. This physiological response is higher after exercise in hypoxia, producing higher basal VO_2_ levels after exercise in hypoxia compared to the same exercise in normoxia [19]. Thus, rugby players may have a greater baseline VO_2_ at the beginning of the RSA test when performing an AH re-warm-up. This finding is supported by the lower values of blood lactate concentration obtained by the players after AH compared with AN and RN.

The inclusion of an AH re-warm-up strategy during an inactive phase after warm-up enhanced CMJ and sprint performance when compared with an RN protocol. One possible mechanism behind the enhanced sprinting and jumping performance after AH re-warm-up is PAP. This phenomenon enhances motor unit excitability, creating an improved ability for power production [30]. However, in the present study, PAP did not occur after AN, and one may argue that it does not explain the superior performance after active R-WU strategies (AH and AN) in comparison to rest conditions (RH and RN). However, it has been previously reported that exercise in hypoxia produces a higher motor unit recruitment [21] than the same exercise in normoxia, and for this reason, the PAP may be larger after AH than AN. Therefore, the inclusion of AH during an inactive phase after warm-up could increase the subsequent jump and sprint performance in rugby players.

It has been suggested that attenuation of the impairment of muscle temperature by the application of an R-WU strategy (such as the use of a passive heat jacket and/or the inclusion of active exercises) [7,24] optimizes sprint and jump performance in rugby players more than passive rest. This may be related to some physiological effects that are highly dependent on an increase in body temperature, such as an increase in nerve conduction rate [5], increased blood flow and the optimization of metabolic reactions [3], or reduced muscle and joint resistance [2]. These physiological effects could explain the data obtained in the present study, where in comparison with the two passive rest trials (RH and RN), a significantly higher change from baseline Ttymp was found after AH and AN. In addition, a concomitant increase in sprint, jump and RSA performance was reported after AH but not after AN. These divergent performance results between AN and AH could be related to the higher values of Ttymp obtained in AH than in AN, and with the hypoxic but non-dependent body temperature effects described above (i.e., VO_2_ kinetics, muscle perfusion and metabolic effects).

The main limitation of the present study was the small sample size analysed. In addition, it is necessary to analyse the effect of this type of strategy using other types of R-WU task (e.g., sprint exercises) because the stress of the task selected in the present study may not be stressful enough. Another limitation may be related to the procedures prior to the R-WU. Since there is no possibility of taking our equipment to the rugby field during an official match, before the application of the R-WU protocols, we prepared a sequence of tests that, according to our experience, would allow, in physiological terms, to simulate the maximum possible a real game situation. However, these tests, when carried out in a shorter period of time (15 min), may not be enough to obtain the desired physiological adaptations; therefore, these tests may eventually be rethought in the future in order to bring the context even closer real. In terms of the methodological procedures employed herein, the fact that some physiological and metabolic variables were not assessed (i.e., VO_2_, muscle and core temperature, electromyography, etc.) may also be considered a potential limitation. This research unveils a new line of re-warm-up protocols under hypoxic conditions aimed at maximizing performance after an inactive phase or during half-time in team sports. From an applied perspective, coaches should bear in mind that if the inactive phase between warm-up and competition is too long, the inclusion of active exercise under hypoxia is a suitable way to minimize decremental effects on performance that long periods of inactivity can produce. Knowing that it is a device that involves some costs, its applicability in amateur teams may not be possible, but in professional and national teams, which have some financial health, the use of hypobaric chambers can result in a strategy aimed at optimization of the performance of its players.

## 5. Conclusions

The application of an active re-warm-up routine under hypoxic conditions for a 15-min period after warm-up optimized a 20 m sprint test, CMJ performance and mean RSA performance in professional rugby players. Knowing that it is a device that involves some costs, its applicability in amateur teams may not be possible, but in professional and national teams, which have some financial health, the use of hypobaric chambers can result in a strategy aimed at optimization of the performance of its players.

## Figures and Tables

**Table 1:** Results during warm-up and post-warm up in the four strategies applied.

				95% Confidence Interval		ANOVA		
		Mean	SD	Lower	Upper	*F*	*p*	*η* ^2^
Baseline Ttymp (°C)	RN	36.35	0.48	36.00	36.70	0.228	0.874	0.089
	AN	36.26	0.44	35.95	36.57			
	RH	36.22	0.28	36.02	36.42			
	AH	36.21	0.39	35.93	36.49			
Mean HR WU (bpm)	RN	138.70	20.76	123.85	153.55	0.995	0.449	0.299
	AN	141.60	19.58	127.59	155.61			
	RH	139.60	17.09	127.38	151.83			
	AH	138.20	16.98	126.05	150.35			
Peak HR WU (bpm)	RN	171.90	9.92	164.81	178.99	0.542	0.668	0.189
	AN	170.70	10.06	163.51	177.89			
	RH	171.70	8.69	165.48	177.92			
	AH	172.00	11.24	163.96	180.04			
RPE WU (AU)	RN	5.50	1.65	4.32	6.68	1.007	0.444	0.302
	AN	5.40	1.96	4.00	6.80			
	RH	5.80	1.87	4.46	7.14			
	AH	6.00	1.41	4.99	7.01			
20 m during WU (s)	RN	3.63	0.30	3.42	3.85	1.631	0.267	0.411
	AN	3.67	0.36	3.42	3.93			
	RH	3.51	0.38	3.24	3.78			
	AH	3.62	0.33	3.38	3.85			
30 m during WU (s)	RN	6.15	0.37	5.89	6.42	0.604	0.633	0.206
	AN	6.22	0.38	5.95	6.49			
	RH	6.17	0.46	5.84	6.49			
	AH	6.04	0.41	5.75	6.33			
CMJ height post WU (cm)	RN	38.26	5.90	34.04	42.48	1.382	0.325	0.372
	AN	37.30	5.09	33.66	40.94			
	RH	36.58	4.12	33.63	39.53			
	AH	37.00	5.10	33.35	40.65			

RN: rest in normoxia, AN: activity in normoxia; RH: rest in hypoxia; AH: activity in hypoxia; HR: heart rate; RPE: rating of perceived exertion; AU: arbitrary units; WU: warm-up; CMJ: countermovement jump; Ttymp: the tympanic temperature.

**Table 2:** Results during the 15 min period after warm up in the four re-warm-up strategies applied.

				95% Confidence Interval		ANOVA			Post-Hoc	
		Mean	SD	Lower	Upper	*F*	*p*	*η* ^2^	Comparison	*p*
SaO_2_ (%)	RN	97.50	0.53	97.12	97.88	30.443	<0.001	0.929	RN vs. RH	<0.001
	AN	97.30	0.82	96.71	97.89				RN vs. AH	<0.001
	RH	90.80	2.10	89.30	92.30				AN vs. RH	<0.001
	AH	87.90	4.20	84.89	90.91				AN vs. AH	<0.001
Mean HR (bpm)	RN	95.40	15.95	83.99	106.81	1.424	0.314	0.379		
	AN	103.40	13.89	93.46	113.34					
	RH	102.30	18.66	88.96	115.65					
	AH	106.50	12.73	97.39	115.61					
Peak HR (bpm)	RN	131.60	22.00	115.87	147.34	2.574	0.137	0.524		
	AN	145.00	12.95	135.73	154.27					
	RH	131.20	25.84	112.72	149.68					
	AH	150.90	17.22	138.58	163.22					
Ttymp post RWU (Δ from baseline)	RN	−0.18	0.36	−0.43	0.08	7.007	0.016	0.750	RN vs. AN	0.031
	AN	0.37	0.21	0.22	0.52				RN vs. AH	0.023
	RH	−0.04	0.20	−0.18	0.10				RH vs. AN	0.005
	AH	0.47	0.31	0.25	0.69				RH vs. AH	0.015

RN: rest in normoxia, AN: activity in normoxia; RH: rest in hypoxia; AH: activity in hypoxia; HR: heart rate; SaO_2_: arterial oxygen saturation; RWU: re-warm-up; Ttymp: the tympanic temperature.

**Table 3:** Results after the 15 min period after warm-up in the four re-warm-up strategies applied.

				95% Confidence Interval		ANOVA			Post-Hoc	
		Mean	SD	Lower	Upper	*F*	*p*	*η* ^2^	Comparison	*p*
CMJ height post R-WU (cm)	RN	34.29	4.55	31.04	37.54	4.961	0.037	0.680	RN vs. AH	0.01
	AN	34.56	4.36	31.45	37.68					
	RH	34.52	5.66	30.47	38.57					
	AH	35.78	4.69	32.43	39.13					
20 m post R-WU (s)	RN	3.89	0.36	3.63	4.15	6.492	0.02	0.736	RN vs. AH	<0.001
	AN	3.77	0.30	3.56	3.98					
	RH	3.78	0.35	3.53	4.03					
	AH	3.64	0.28	3.44	3.84					
Mean HR RSA (bpm)	RN	162.70	7.66	157.22	168.18	0.864	0.503	0.270		
	AN	161.10	15.29	150.16	172.04					
	RH	165.50	11.95	156.95	174.05					
	AH	165.70	12.45	156.80	174.60					
Peak HR RSA (bpm)	RN	179.30	4.99	175.73	182.87	1.316	0.343	0.361		
	AN	177.30	7.27	172.10	182.50					
	RH	180.00	7.80	174.42	185.58					
	AH	181.30	10.20	174.00	188.59					
Blood lactate (mMol/l)	RN	18.59	6.47	13.96	23.22	9.856	0.007	0.809	RN vs. AH	0.015
	AN	15.53	5.87	11.33	19.73				AN vs. AH	0.036
	RH	13.28	4.26	10.24	16.33					
	AH	9.96	1.64	8.79	11.13					
RPE RSA (AU)	RN	7.60	1.35	6.63	8.57	1.681	0.257	0.419		
	AN	7.70	1.16	6.87	8.53					
	RH	7.70	0.95	7.02	8.38					
	AH	8.40	1.35	7.43	9.37					
Best RSA (s)	RN	6.08	0.35	5.82	6.33	2.791	0.119	0.545		
	AN	6.04	0.34	5.79	6.28					
	RH	6.04	0.37	5.78	6.31					
	AH	5.98	0.33	5.75	6.22					
Mean RSA (s)	RN	6.27	0.38	6.00	6.54	9.143	0.008	0.797	RN vs. AH	0.02
	AN	6.24	0.34	5.99	6.48				RH vs. AH	0.02
	RH	6.27	0.36	6.01	6.53					
	AH	6.19	0.32	5.96	6.42					
Fatigue Index RSA test (%)	RN	3.14	1.59	2.00	4.27	0.465	0.716	0.166		
	AN	3.34	0.90	2.70	3.99					
	RH	3.85	1.45	2.81	4.89					
	AH	3.43	1.31	2.49	4.36					
Mean SmO_2_ RSA (%)	RN	42.98	20.65	28.21	57.75	6.269	0.021	0.729	RN vs. AH	<0.01
	AN	42.32	18.50	29.09	55.55				RH vs. AH	0.01
	RH	42.27	18.08	29.33	55.20				AN vs. AH	0.01
	AH	37.19	16.03	25.72	48.65					
Mean muscle Hb RSA (mg/dl)	RN	12.05	0.59	11.63	12.48	4.685	0.042	0.668	RN vs. AH	0.05
	AN	12.17	0.54	11.78	12.56					
	RH	12.11	0.79	11.54	12.67					
	AH	12.40	0.57	11.99	12.81					
Tª post RSA (Δ from baseline)	RN	0.26	0.52	−0.11	0.63	0.477	0.708	0.170		
	AN	0.14	0.96	−0.55	0.83					
	RH	0.15	0.47	−0.18	0.48					
	AH	0.14	0.39	−0.14	0.42					

RN: rest in normoxia, AN: activity in normoxia; RH: rest in hypoxia; AH: activity in hypoxia; Hb: haemoglobin; HR: heart rate; R-WU: re-warm-up; RPE: rating of perceived exertion; AU: arbitrary units; RSA: repeat sprint ability test; SmO_2_: muscle oxygen saturation; CMJ: countermovement jump Tª: temperature.
